# Pharmacy Practice and Education in Finland

**DOI:** 10.3390/pharmacy7010021

**Published:** 2019-02-23

**Authors:** Jouni Hirvonen, Outi Salminen, Katariina Vuorensola, Nina Katajavuori, Helena Huhtala, Jeffrey Atkinson

**Affiliations:** 1 Faculty of Pharmacy, University of Helsinki Viikinkaari 5 E, PO Box 56, 00014 Helsinki, Finland; jouni.hirvonen@helsinki.fi (J.H.); outi.salminen@helsinki.fi (O.S.); katariina.vuorensola@helsinki.fi (K.V.); Helena.huhtala@helsinki.fi (H.H.); 2 Centre for University Teaching and Learning, University of Helsinki, Viikinkaari 11, P.O.Box 62, 00014 Helsinki, Finland; nina.katajavuori@helsinki.fi; 3 Pharmacolor Consultants Nancy, 12 rue de Versigny, 54600 Villers, France

**Keywords:** pharmacy, education, practice, Finland, European Union

## Abstract

The Pharmacy Education in Europe (PHARMINE) project studies pharmacy practice and education in the European Union (EU) member states. The work was carried out using an electronic survey sent to chosen pharmacy representatives. The surveys of the individual member states are now being published as reference documents for students and staff interested in research on pharmacy education in the EU, and in mobility. This paper presents the results of the PHARMINE survey on pharmacy practice and education in Finland. Pharmacies have a monopoly on the dispensation of medicines. They can also provide diagnostic services. *Proviisori* act as pharmacy owners and managers. They follow a five-year (M.Sc. Pharm.) degree course with a six-month traineeship. *Farmaseutti*, who follow a three-year (B.Sc. Pharm.) degree course (also with a six-month traineeship), can dispense medicines and counsel patients in Finland. The B.Sc. and the first three years of the M.Sc. involve the same course. The current pharmacy curriculum (revised in 2014) is based on five strands: (1) pharmacy as a multidisciplinary science with numerous opportunities in the working life, (2) basics of pharmaceutical sciences, (3) patient and medication, (4) optional studies and selected study paths, and (5) drug development and use. The learning outcomes of the pharmacy graduates include (1) basics of natural sciences: chemistry, physics, technology, biosciences required for all the students (B.Sc. and M.Sc.), (2) medicine and medication: compounding of medicines, holism of medication, pharmacology and biopharmaceutics (side-effects and interactions), patient counseling, efficacy and safety of medicines and medication, (3) comprehensive and supportive interactions of the various disciplines of pharmacy education and research: the role and significance of pharmacy as a discipline in society, the necessary skills and knowledge in scientific thinking and pharmaceutical research, and (4) basics of economics and management, multidisciplinarity, hospital pharmacy, scientific writing skills, management skills. In addition, teaching and learning of “general skills”, such as the pharmacist’s professional identity and the role in society as a part of the healthcare system, critical and creative thinking, problem-solving skills, personal learning skills and life-long learning, attitude and sense of responsibility, and communication skills are developed in direct association with subject-specific courses. Professional specialization studies in industrial pharmacy, and community and hospital pharmacy are given at the post-graduate level at the University of Helsinki.

## 1. Introduction

The Pharmacy Education in Europe (PHARMINE) consortium surveyed pharmacy practice and education in the member states of the European Union (EU), including Finland, between 2008 and 2011, with an update in November 2018. The methodology used in the PHARMINE study and the main results obtained were already published [1]. In the first part of the study, PHARMINE gathered information on community practice, and on specialized hospital and industrial practice, as well as the necessary education and training. PHARMINE also dealt with other personnel working in pharmacies such as pharmacists’ assistants: their education, training, and responsibilities.

PHARMINE went on to study the legal and administrative context of pharmacy practice and education. In the EU, pharmacy practice and education fall under two jurisdictions: European and national. EU legislation is confederal in structure. Freedom of movement and of exercise of profession is the cornerstone. To ensure this, there is a system of automatic recognition of professional qualifications for sectoral professions such as not only pharmacists, but also nurses, midwives, doctors, dentists, architects, and veterinary surgeons. To work in another EU member state, such professionals apply to the relevant authority of that country, providing proof of the qualifications obtained in their home state. Such procedures are regulated by directives issued by the European Commission of the EU. The latter are ordinances laying down the broad imperatives on the practice and education of the given profession [2]. An EU directive requires member states to achieve a particular result—in this case, harmonization of practice and education—without dictating the means of achieving that result. Thus, directives leave the different member states with leeway as to the exact rules to be adopted. The result of this is that member states have systems that are more or less harmonized with the EU paradigm. 

In parallel to the above pan-national system, member states may introduce national legislation relating for example to specialized practice, and to ownership and management of pharmacies. 

Pharmacy education and training in Europe is also influenced by the Bologna agreement on the harmonization of European degree courses, and student and staff exchange [3]. The Bologna agreement was signed by the education ministers of the governments of the European Higher Education Area (48 members including the 28 EU member states). It proposes recommendations that are not legally binding. The first of these is a harmonized structure for all university degrees (including pharmacy) with a bachelor (three years) followed by a master (two years) degree. In this aspect, the Bologna agreement is in opposition to the EU directive. The latter requires a five-year, “tunnel” degree structure for pharmacy, i.e., a degree course that offers no possibility for intermediate entry or exit after accomplishment of a three-year bachelor period. 

The idea behind the Bologna recommendations is to improve student mobility, with the development of tools to promote student exchange programs like the European Credit Transfer and Accumulation System (ECTS). This provides credits to students for defined learning outcomes. ECTS are coupled to a Diploma Supplement that describes the nature, level, context, content, and status of the studies that were successfully completed by a student. This system allows students to validate studies carried out at their host university by their home university. 

This paper looks at how the EU directive and the Bologna recommendations apply to Finland, a country that has been a member of the EU for quite some time (membership 1995). 

In order to place practice within the general health situation in Finland compared to Europe, it can be noted that Finland has a compulsory, tax-based healthcare system, which provides comprehensive coverage for the entire resident population. The central government and municipalities are the main players in the organization of healthcare. At the national level, the Ministry of Social Affairs and Health issues framework legislation on health and social care policy and monitors implementation. At the local level, the municipal health committee, council, and executive board make decisions on the planning and organization of care. Municipalities (317 in 2016) are also responsible for health promotion and disease prevention, primary medical care, medical rehabilitation, and dental care. The country is divided into 20 hospital districts, each of which is a federation of municipalities responsible for arranging and coordinating specialized care within their area. There is an ongoing major reform in health and social care in Finland currently, which will probably cause numerous changes in arrangements and responsibilities in the area [4]. 

In 2015, the total healthcare expenditure in Finland was €19.8 billion, with an annual growth of 1.2%. This means 9.4% of gross domestic product. Specialized medical care costs were €6.9 billion and basic medical care costs were €3.7 billion. Drug costs and medical supplies were €2.5 billion (+4.0% compared to 2014). Public funding covered 74.6% (−1% compared to 2014) and private funding covered 25.4% of healthcare costs in Finland in 2016 [5]. Pharmaceutical care is an integral part of social and healthcare. The Ministry of Social Affairs and Health is responsible for the development of pharmaceutical care and preparing legislation concerning pharmaceuticals. The aim is to have an efficient, safe, appropriate, and cost-effective system of medical treatment available to all who need it. The proper availability and professionally competent distribution of pharmaceuticals is safeguarded at all times.

## 2. Design

Information was obtained from academics and practicising pharmcists (the authors) and from internet sources (university websites) on the following:

- pharmacy:
  ○ practice (community, hospital, and industrial);
  ○ legislation;
  ○ education and training;
- harmonization with the EU sectoral directive on pharmacy [2] and with the Bologna recommendations [6].

Electronic survey methodology was used; data were collected in 2010 and revised in 2017–2018. Sampling was performed by sending the survey to all pharmacy departments, pharmacy orders and chambers, associations of industrial, hospital, and other specialization pharmacists, and associations of pharmacy students. Collection of data in any specific country took between six and 12 months. Data collection was performed electronically using standard survey platforms. We attempted at all times to collect objective, if possible, numerical data.

The information is presented in the form of tables in order to facilitate legibility. This presentation was developed in association with the Pharmacy journal editorial board; it is based on the organization of the PHARMINE survey [1], and was described in detail in a previous publication [7]. This format will ease the comparison of different EU countries by students and staff envisaging exchange programs, and by researchers in pharmacy education and practice.

## 3. Evaluation and Assessment

In order to follow the terminology of Finnish pharmacy degrees, three major personnel categories can be separated from each other as follows:

Pharmacy personnel in Finland

M.Sc. Pharm. (*Proviisori*) = Pharmacists who dispense medicine and counsel patients; the degree is a requirement for pharmacy owners and managers—a five-year university education.

B.Sc. Pharm. (*Farmaseutti*) = Pharmacists who dispense medicine and counsel patients in pharmacies but cannot own a pharmacy or be a head manager in a pharmacy—a three-year university education.

Pharmacy technician (*Lääketeknikko*) = Supporting personnel in pharmacies taking care of medicine storage, logistics, invoicing, and cash services—a 2–3-year upper secondary vocational education.

### 3.1. Organization of the Activities of Pharmacists, Professional Bodies

Table 1 provides details of the numbers and activities of community pharmacists and pharmacies in Finland. Items are expounded in the “comments” column.

Using the data in Reference [1] and Table 2, it can be calculated that, compared to the EU linear regression estimation (for definition and calculation, see Reference [1]), the ratio of the number of community pharmacists in Finland to the population compared to the linear regression estimation = 0.40. Thus, the number of pharmacists per population is lower than the EU norm. The same comparison for community pharmacies produces a ratio of 0.5, lower than the EU norm. This may reflect the separate activities of pharmacists compared to other healthcare professionals.

The activities and occupations of pharmacists in Finland are similar to those of community pharmacists in other EU member states [1]. 

Table 2 provides details of the numbers and activities of persons other than pharmacists working in pharmacies in Finland.

Finland is one of the rare if not the only country in the EU which follows the Bologna principles to the letter. Thus, there are separate three- (B.Sc. Pharm.) and five-year (M.Sc. Pharm.) degrees each with a job profile following graduation. This topic is discussed in detail later.

Table 3 provides the numbers and activities of hospital pharmacists in Finland.

The number of pharmacists working in hospitals is lower than the EU average. The ratio of the actual number compared to the linear regression estimation is 1.94, (for definition and calculation, see Reference [1]). The duties of hospital pharmacists are similar to those elsewhere in the EU [1].

Turning to pharmacists in industry and in other sectors, Table 4 provides such information on these sectors in Finland.

Table 5 provides information on professional associations for pharmacists in terms of number, territorial distribution, and ethical and professional attributes in Finland.

### 3.2. Pharmacy Faculties, Students, and Courses

Table 6 provides detailed numbers and activities of pharmacy higher-education institutions (HEIs), staff, and students in Finland.

A comparison to the EU average for staff shows that Finland has a high ratio 2.1 [1], and the number of pharmacy HEIs is also high compared to the EU norm at 1.5. Concerning teaching, it is interesting to note that Finnish pharmacy HEIs offer an implemented Bologna style B.Sc. Pharm. degree.

Table 7 below contains details of specialization electives.

Post-graduate specialized education is offered in industrial pharmacy and in community and hospital pharmacy.

Table 8 provides details of past and present changes in pharmacy education and training in Finland.

### 3.3. Teaching and Learning Methods—Student Hours

Table 9 represents teaching and learning methods in student hours.

The first year is devoted mainly to lectures and the second and third years are devoted to traineeship.

### 3.4. Subject Areas

Table 10 provides details of subject area (for definitions of the subject areas, see Reference [1]). 

The hours calculated in every column represent the time scheduled for lectures, assignments, and group works. The time a student takes for individual work is not calculated here. Students also have to take 20 ETCS of elective studies for B.Sc. These hours were not calculated here, as the hours spent vary for each student and may even be on non-pharmaceutical subjects. 

**Figure 1:**
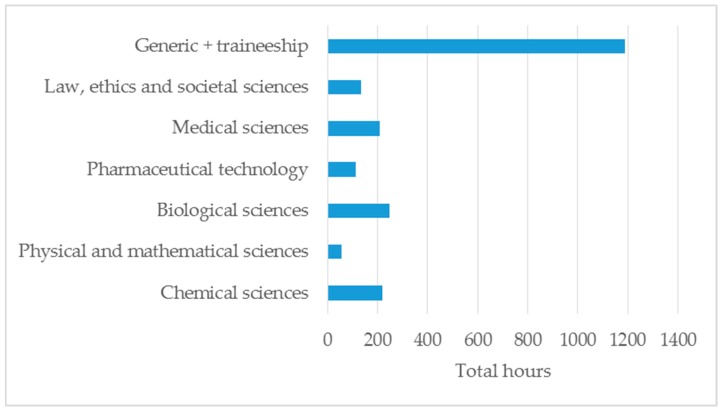
A graphical representation of total hours over the studies of the first three years (B.Sc.) for the various subject areas.

Figure 1 shows a graphical representation of the above.

Year 1 is devoted mainly to chemical and medical sciences, year 2 is devoted to generic subjects and pharmaceutical technology, year 3 is devoted to generic subjects and medical sciences, and year 4 is devoted to chemical sciences and pharmaceutical technology. It can be seen that the MEDISCI/CHEMSCI ratio in the master program (year 4 and 5) is 2.83, thus reflecting the importance of medicinal science subjects. The overall ratio of MEDISCI/CHEMSCI (years 1–5) is 0.73. In the EU, some HEIs such as Spain have a “balanced” course with a medicinal sciences/chemical sciences index of 1.2. Others have more “medical” courses, such as Ireland and the Netherlands, with indices of 2.6 and 1.6, respectively [1].

### 3.5. Impact of the Bologna Principles [3]

Table 11 provides details the various ways in which the Bologna declaration impacts on the pharmacy HEIs of Finland.

Finland adopted the Bologna system with a two-tier degree system, the use of ECTS, etc.

The number of Erasmus and other exchange students increased steadily over the past 5-10 years.

### 3.6. Impact of European Union (EU) Directive 2013/55/EC [2]

Table 12 provides details on the various ways in which the EC directive impacts on pharmacy education and training in Finland.

Finland conforms to the different aspects of the EU directive with a five-year degree and a six-month traineeship. Suitable balance between theoretical studies and practical excercises is to be found in order to reach the curriculum objects and learning outcomes of the B.Sc. and M.Sc. degrees.

## 4. Discussion and Conclusions

We developed pharmacy education in Finland based on teacher and student feedback, and also based on the field-specific feedback from societal stakeholders including community pharmacists, hospital pharmacists, industrial pharmacists, and drug authority experts, and also according to the recommendations and suggestions of the EAFP (European Association of Faculties in Pharmacy)-based PHARMINE and PHAR-QA projects. One main point (but not the only point) is the close correspondence of the Finnish system to the spirit of the essential points of the original Sorbonne 1998 declaration [3], as follows:

- University education will have
  ○ a first-cycle degree (undergraduate, three years) with “international recognition of the first cycle degree as an appropriate level…”, providing the qualifications needed for immediate employment.
  ○ the above will be followed with a Master degree (M.Sc., two years), and eventually a Ph.D. degree (four years)
- Qualifications in both cycles can be obtained in several EU countries; in an “extreme” case (going beyond the typical case of a semester spent abroad), a foreign B.Sc. could be accepted as a requirement for acceptance into the Finnish M.Sc. program.

The main conclusions/implications to be drawn from this survey of pharmacy education and training in Finland are that it follows EU directives and additionally provides a description of the two-tier Sorbonne/Bologna B.Sc./M.Sc. model. 

Possible biases in our study could involve flaws in the representativity of the survey methodology. However, given that Finland is a relatively small EU country with but three pharmacy departments (Helsinki, Kuopio, Turku), we estimate that such biases are of little importance.

## Figures and Tables

**Table 1:** Numbers and activities of Finnish community pharmacists and pharmacies.

Item	Number	Comments.
Community pharmacists	772 M.Sc. pharmacists + 594 pharmacy owners = 1366	There are additionally 3724 bachelor-level pharmacists working in community pharmacies. The total number of employees in community pharmacies is circa 8500.
Community pharmacies	610 + 200 = 810	610 pharmacies and 200 subsidiary or branch pharmacies—the same medicines and services are available from both types of pharmacies. There are approximately 1 pharmacist (M.Sc.) and 4.5 bachelor pharmacists per pharmacy, and 6600 inhabitants per pharmacy.
Competences and roles of community pharmacists		Pharmacists work as the following: dispensing staff members pharmacy owners, managers, specialist pharmacists (after professional post-gradute studies). Competences include administrative issues, customer service, medication review, marketing, education of pharmacy staff, and multidisciplinary co-operation with other healthcare professionals. Pharmacists provide services to help patients monitor the therapeutic control of blood sugar or blood pressure.
Ownership limited to pharmacists?	Yes	A license to own a pharmacy is granted to a person having a 5-year degree in pharmacy with a 6-month traineeship (M.Sc. Pharm.).
Are there rules governing the geographical distribution of community pharmacies? [8]	Yes	The location of community pharmacies is based on the decision made by the Finnish Medicine’s Agency (FIMEA) (https://www.fimea.fi/web/en). FIMEA evaluates if there is a need for one (or multiple) community pharmacies in some particular area and specifies also the area where the pharmacy or pharmacies should be located. Within that specific area, pharmacies are free to choose their exact location. This system assures equal accessibility to medicines and pharmacy services for the whole population.
Are drugs and healthcare products available to the general public by other channels?	No	In Finland, medicines are sold to the public only from pharmacies, with the exception that nicotine replacement therapy (NRT) products may also be available in grocery shops. However, many pharmacies offer internet shop alternatives for healthcare products and over-the-counter drugs. Veterinary drugs are also available from veterinarians.

**Table 2:** Numbers and activities of other personnel working in pharmacies in Finland.

Item	Number	Comments
Are persons other than pharmacists involved in community practice?	Yes	These are pharmacy technicians. Only persons with either a B.Sc.Pharm. or a M.Sc.Pharm. degree are allowed to dispense and counsel patients on medicines. A pharmacist (M.Sc.degree) is responsible for the operation of the pharmacy.
Their titles and number(s)	3486	Pharmacy technicians with upper secondary vocational education (corresponds to “pharmacy assistants”)
Their qualifications		
Organization providing and validating the education and training.		Upper secondary vocational education
Duration of studies	2–3 years	
Subject areas		Pharmacy technicians study logistics, accounting, and information technology (IT) skills. Education consists of theoretical studies and a larger part of in-house training.
Competences and roles		Pharmacy technicians: Their main task is to take care of medicine storage and logistics in the community pharmacy. They also take care, for example, of invoicing and operating of pharmacy IT systems.

**Table 3:** Numbers and activities of hospital pharmacies and pharmacists in Finland.

Item	Number	Comment
Number of hospital pharmacists	696	605 (B.Sc.) + 91 (M.Sc.)
Number of hospital pharmacies	154	There are 24 hospital pharmacies that are in central hospitals and 81 medicine centers which are in other hospitals or healthcare centers. University hospitals are the largest hospitals in Finland. There are five university hospitals that are located in the bigger cities (Helsinki, Tampere, Turku, Oulu, and Kuopio: in cities where there is a university with a medical faculty). Central hospitals are the most central and larger hospitals in some other particular hospital districts. Each central hospital is under the supervision of a given university hospital.
Competences and roles of hospital pharmacists		In most hospitals, the hospital pharmacy or the medicine center is one of the medical service departments. The manager of a hospital pharmacy is required to have an M.Sc. in pharmacy, while the manager of a medicine centre is required to have an M.Sc. or B.Sc. in pharmacy. A manager of a hospital pharmacy or a dispensary is usually authorized by the medical director of the hospital. B.Sc. and M.Sc. hospital pharmacists used to have a logistic role in hospitals and healthcare centers. The role is now starting to change, and some pharmacists are working in the wards. A professional post-graduate specialization program for hospital pharmacists started in 2010 to ensure stronger competencies for the hospital pharmacists to work as clinical specialists (see more details of the specialized education in Table 7).

**Table 4:** Pharmaceutical industry, and numbers and activities of pharmacists in industry and in other sectors in Finland.

Pharmaceutical and Related Industries		
Item	Number	Comment
Number of companies with production, research and development (R&D) and distribution	4	Pharmaceutical production: 1598 million € (m€) Pharmaceutical exports: 852 m€; imports: 2010 m€ (balance −1158 m€) Research and development: 172 m€ Employment in the pharmaceutical industry: 5233 Pharmaceutical market value: 2246 m€ Share of generics in market sales: 25% The above figures are from *The Pharmaceutical Industry in Figures*, European Federation of Pharmaceutical Industries and Associations, EFPIA, Key figures 2017 [9].
Companies with production only	3	
Companies with distribution only	2	
Number of pharmacists working in industry	400 M.Sc. and 400 B.Sc.	
**Other sectors**		
Number of pharmacists working in other sectors	320	This information is based on the report by Akava—Confederation of Unions for Professional and Managerial Staff in Finland in 2008 [10].
Sectors in which pharmacists are employed		Academic sector, e.g., pharmacists working in universities and research organizations (160) Administration, e.g., pharmacists working in Finnish national authorities (Finnish Medicines Agency, Ministry of Social Affairs and Health, National Insurance Institution) (60) Other: unspecified (100)
Competences and roles of pharmacists employed in other sectors		Teaching, research, administration, management, and leadership Varying roles and competencies: specialist pharmacists (pharmacists specialized in some specific issues, for example, marketing authorizations, pricing and re-imbursement of medical products, IT-issues such as e-prescriptions and databases, medicine information), researchers, managers

**Table 5:** Professional associations for pharmacists in Finland.

Item	Reply	Comments
Registration of pharmacists	Yes. There are circa 2000 registered pharmacists in Finland.	Issued by Valvira (National supervisory authority for Welfare and Health) [11].
Creation of community pharmacies and control of territorial distribution	Yes	Issued by Finnish Medicines Agency (FIMEA) [8]. From the FIMEA website: Under Section 40 of the Medicines Act, the operation of a pharmacy business requires a licence (pharmacy licence) issued by FIMEA. The conditions under which a pharmacy licence may be granted according to the Section 43 of the Medicines Act are as follows: “A pharmacy licence may be granted to citizen of a European Economic Area state who is a certified Master of Pharmacy and who has not been declared bankrupt or legally incompetent or who has not been assigned a person to supervise his or her interests. If there is more than one applicant, a pharmacy licence is granted to the applicant who can be considered to have the overall best potential for operating a pharmacy business. In assessing the potential, the applicant’s work in pharmacies and other pharmaceutical services and studies, managerial skills, and other activities pertinent to operating a pharmacy business must be taken into account.”
Ethical considerations and role of pharmacists in healthcare	Yes	There is an advisory board on ethical issues in pharmacies based on the co-operation between Pharmacy Owners’ Association (AFP [12]) and Finnish Pharmacists’ Association (SFL [13]). Additionally, there exists a national ethical code of conduct produced by abovementioned organizations. In order to strengthen the role of community pharmacies in healthcare and to support the professional development, the Association of Finnish Pharmacists established a national strategy in 1997 that concerned pharmacy services and pharmacy role in healthcare. This strategy highlighted the importance of medication counseling in community pharmacies: whenever medicines are dispensed, information should also be provided. National long-term programs focusing on chronic diseases (asthma, diabetes, and heart diseases) were organized to encourage local co-operation between pharmacies and other healthcare professionals and to develop the competency and counseling skills of pharmacy staff.
Quality assurance and validation of higher-education institution (HEI) courses for pharmacists	No	The universities providing pharmacy education have their own quality handbooks and quality assurance procedures. In the University of Helsinki, for example, feedback is collected from students and internal and external/international audits are made regularly.

**Table 6:** Pharmacy higher-education institutions (HEIs), staff, and students in Finland.

Item	Reply and/or Number	Comments
Total number of HEIs for pharmacy	3	Helsinki, Kuopio, Turku. In total, there are 14 universities and 23 universities of applied sciences in Finland.
Public HEIs	3	
**Organization of HEIs**		
Independent faculty	Yes	University of Helsinki, Faculty of Pharmacy
Attached to a medical faculty	Yes	University of Eastern Finland, Faculty of Health Sciences, in Kuopio
Attached to a science faculty	Yes	Åbo Akademi University, Faculty of Mathematics and Natural Sciences, in Turku
Do HEIs offer B.Sc. and M.Sc. degrees?	Yes	Universities of Helsinki and Eastern Finland
Do HEIs offer a M.Sc. Pharm. after a B.Sc. degree in another discipline?	Yes	
**Finland**		
**Teaching staff**		
Number of teaching staff (from Finland)		circa 260
Number of international teaching staff (from European Union (EU) MSs)		circa 30
Number of international teaching staff (non-EU)		circa 20
Number professionals (pharmacists and others) from outside the HEI, involved in education and training.		circa 50
**Students**		
Places on entry after secondary school	350 + 110	The numbers are rounded. All students follow the same course for the first 3 years with 350 having the right to study only B.Sc. Pharm. (and taking up employment after graduating), and 110 going on for two further years to a M.Sc. Pharm.
Number of applicants for entry	3000	Approximately 6 applicants for 1 place.
Number that become professional pharmacists.	350 B.Sc. + 110 M.Sc.	Around 350 take up employment after graduating B.Sc. and do not have the right to continue with the M.Sc. course. Around 110 continue with the M.Sc. course.
Number of international students (from EU member states (MS))	circa 75	Exchange students, mostly M.Sc. students
Number of international students (non-EU)	circa 20	Exchange students, mostly M.Sc. students
**Entry requirements following secondary school (national)**		
Specific pharmacy-related, national entrance examination	Yes	The same national entrance examination in pharmacy is used in all HEIs.
Is there a national numerus clausus?	No	Each institution sets its individual numerus clausus.
**Advanced entry**		
At which level?		Some of the students have the right to pursue both B.Sc. and M.Sc. (Pharm.) degrees. Persons taken the B.Sc. (Pharm.) degree can apply to the Master’s level also later.
Specific requirements for international students (EU or non-EU).		Language requirements in Finnish or Swedish for B.Sc. and M.Sc.
**Fees per year**		
For home students	0	There are no tuition fees for national or international B.Sc. and M.Sc. (Pharm.) degree students.
For EU MS students	0	
For non-EU students	0	
**Helsinki**		
**Teaching staff**		
Number of teaching staff (from Finland)		circa 120
Number of international teaching staff (from EU MSs)		circa 20
Number of international teaching staff (non-EU)		circa 20
Number professionals (pharmacists and others) from outside the HEIs, involved in education and training.		circa 25
**Students**		
Places on entry after secondary school	143 + 60	All students follow the same course for the first 3 years with 143 having the right to study only B.Sc. Pharm. (and taking up employment after graduating), and 60 going on for two further years to a M.Sc. Pharm.
Number of applicants for entry	1500	Approximately 7 candidates per place.
Number becoming professional pharmacists.	150 + 50	
Number of international students (EU)	50	Exchange students, mostly M.Sc. students
Number of international students (non-EU)	10	Exchange students, mostly M.Sc. students
**Entry requirements following secondary school**		
HEI has a specific pharmacy-related entrance examination	Yes	The same national entrance examination in pharmacy is used in all HEIs.
**Advanced entry**		
At which level?		Some of the students have the right to pursue both B.Sc. and M.Sc. (Pharm.) degrees. Persons taken the B.Sc. (Pharm.) degree can apply to the Master’s level also later.
Specific requirements for international students (EU/non-EU).		Language requirements in Finnish or Swedish for B.Sc. and M.Sc. degrees [14]. For exchange students no specific language requirements. The courses taught in English in Helsinki are listed in Reference [15].
**Fees per year**		Free for all B.Sc. and M.Sc. (Pharm.) degree students.

**Table 7:** Specialization electives in pharmacy HEIs.

Item	Reply and/or Number	Comment
Does your HEI provide specialized courses?	Yes	
In which years?		Both after completing the B.Sc. and the M.Sc. degree
In which specialization (industry, hospital…)?		1. Industrial pharmacy 2. Community and hospital pharmacy Specialization studies are post-graduate programs for pharmacists (both M.Sc. and B.Sc. Pharm.) working in these specialization areas. Studying takes place alongside work and study plan is tailored individually to support the student’s own development needs. The extent of studies is 60 European Credit Transfer and Accumulation System (ECTS) for M.Sc. (Pharm.) and 40 ECTS for B.Sc. (Pharm.). Normative duration of studies is 3–4 years. The student fee is 6000 € for the M.Sc. graduates in Pharm. and 4000 € for the B.Sc. graduates in Pharm. (ca. 1500 €/year).
What are the student numbers in each specialization?	20	In both specialization programs, the yearly intake of Bachelors and Masters is altogether 20.

**Table 8:** Past and present changes in education and training in Finnish pharmacy HEIs.

Item	Reply	Comment
Major changes before 2019 in Helsinki/Finland?	Yes	Four new positions for tenure track professors were appointed to the Faculty of Pharmacy. The professional specialization education programs were reformed 2016. The curricula for Bachelor’s and Master’s levels were reformed 2014–2017. A new organization was introduced in the university for degree study programs. The leaders and steering groups for the Bachelor and Master study programs took action in 2016.
Major changes envisaged in Helsinki/Finland?	Yes	The implementation of the new curriculum as a whole in the degree programs.

**Table 9:** Teaching and learning methods in student hours.

Method	Year 1	%	Year 2	%	Year 3	%	Year 4 *	Year 5 **
Lecture	427	72	223	22	98	12	160	
Practicals	36	6	189	19	46	6	0	
Project work	70	12	41	4	120	14	95	
*Subtotal*	*533*		*453*		*264*		*255*	
Traineeship Community	0	0	520 (= 13 weeks)	52	520 (= 13 weeks)	62	0	
*Subtotal*	*533*		*973*		*784*		*255*	
Electives: choice	61		28		49			
*Total*	*594*		*1001*		*833*			

* This represents only part of the M.Sc. course of the fourth and fifth years (see Section 4). ** The curriculum for year 5 varies student-specifically, so numbers of hours are not available. Note: “Practical” includes laboratory work and patient counseling practice. Project work includes all group work and excercises together with writing the thesis of B.Sc. (pharmacy) in the third year.

**Table 10:** Subject areas (ECTS).

Subject Area	Year 1	%	Year 2	%	Year 3	%	Year 4 ^§^	Total ^#^
Chemical sciences	10 ECTS 112 h	21	15 ECTS 108 h	11				220 h
Physical and mathematical sciences	3 ECTS 28 h	5	2 ECTS 28 h	3				56 h
Biological sciences	10 ECTS 68 h	13	12 ECTS 135 h	14				203 h
Pharmaceutical technology	5 ECTS 28 h	5	5 ECTS 84 h	8				112 h
Medical sciences	23 ECTS 166 h	32	11 ECTS 64 h	6	4 ECTS 46 h	7		276 h
Law, ethics and societal sciences	7 ECTS 99 h	19			5 ECTS 36 h	5		135 h
Generic subjects	5 ECTS 24 h	5	3 ECTS 56 h	6	10 ECTS 68 h	10		148 h
Subtotal	525 h		475 h		150 h		255 h	1150 h
Generic subjects + traineeship	5 ECTS 24 h	5	18 ECTS 576 h	58	25 ECTS 588 h	88		1188 h
Total	**525 h**		**995 h**		**670 h**		**255 h**	**2445 h**

^§^ Notes for the 4th year studies: in the M.Sc. degree (120 ECTS), the students take 50 ECTS general studies (of which 20 ECTS are elective) and 70 ECTS major studies. The number of hours spent in every subject area in major studies varies from student to student. Due to this, it is difficult to give an average number of hours. The 30 ECTS of general, compulsory studies for every M.Sc. degree student include highly integrated studies in chemical, biological, medical, and societal sciences, together with pharmaceutical technology, pharmacoeconomics, and statistics. Due to the integrated nature of the studies, detailed information about the hours is not available. Therefore, only a sum of the hours of lectures, assignments, and group works is reported here. ^#^ Total hours by subject area are calculated for the years 1–3, the total hours include also the general, compulsory studies (30 ECTS) of fourth year. The percentage values are not informative and, therefore, are not reported here. The studies cover chemical, biological, and medical sciences, together with pharmaceutical technology, quite evenly throughout the first two years, with the emphasis on medical sciences in the first year. The third year is devoted mainly to generic substances together with traineeship. All the subject areas are covered in the studies of the fourth year in a highly integrated manner.

**Table 11:** Ways in which the Bologna declaration impacts on Finnish pharmacy HEIs.

Bologna Principle	Is the Principle Applied?	Comments
1. Comparable degrees/diploma supplement	Yes	Each graduating student receives a diploma supplement in English
2. Two main cycles (B and M) with entry and exit at B level	Yes	We have a 3-year Bachelor and a 2-year Master program according to the Bologna Agreement. In the University of Helsinki, entrance is permitted each year for 150 students (B.Sc.) and 55 students (M.Sc.). Bachelors graduate after 3 years, and Masters graduate after 5 years. It is possible for a person with a B.Sc. (Pharm.) to gain entrance in the M.Sc. (Pharm.) program if passing an entrance exam or on the grounds of good grades in the Bachelor level studies. Bachelors in Pharmacy are employed in Finland and Sweden in community pharmacies, hospital pharmacies, industry, etc. They constitute the main work force in Finnish community pharmacies. In other parts of Europe, the degree is not recognized.
3. ECTS system of credits/links to LLL (life-long learning)	Yes	All our courses are built according to the ECTS system based on a yearly workload of 1600 h. We accept ECTSs obtained in other European countries to the full. Our students get ECTS points for the compulsory traineeship included in their degree. Since the traineeship is 6 months, the points given are 30, i.e., 5/month. All HEIs in Finland use ECTS-based credit points since 2005. The ECTSs gained before and after graduation are comparable.
4. Obstacles to mobility	Yes	The biggest obstacle to student mobility is the strictly organized curriculum, which does not easily allow students to move. If they are willing to prolong their studies by a half or one year, mobility becomes much easier. In reality, this means that most of our exchange students choose to do their Master’s project abroad, because, by this stage in their university career, they have fewer compulsory courses. Language and financial considerations are not major obstacles to mobility.
5. Erasmus staff exchange to HEI from elsewhere	Yes	Number of staff months: 0.25
6. Erasmus staff exchange from HEI to other HEIs	Yes	Number of staff months: 0.25
7. Erasmus student exchange to HEI from elsewhere	Yes	Number of student months: circa 250 All over Europe
8. Erasmus student exchange from HEI to other HEIs	Yes	Number of student months: circa 50

**Table 12:** Ways in which elements of the European Commission (EC) directive (left column) impacts on Finnish pharmacy HEIs.

The Directive States	How Does/Will This Directive Statement Affect Pharmacy Education and Training?
“Evidence of formal qualifications as a pharmacist shall attest to training of at least five years’ duration…”	This statement does not apply to the first-phase B.Sc. degree in Pharmacy. This statement was obviously taken into consideration when the curriculum for the M.Sc. (Pharm.) degree was developed in 2006.
“…four years of full-time theoretical and practical training at a university or at a higher institute of a level recognized as equivalent, or under the supervision of a university”	Master students study 4.5 years at the university, so this requirement is fulfilled.
“…six-month traineeship in a pharmacy which is open to the public or in a hospital, under the supervision of that hospital’s pharmaceutical department”	Both Bachelor and Master students perform the six-month traineeship. At least three months have to be spent in a community pharmacy and the remaining three months can be spent in a community or hospital pharmacy. The first three months of traineeship are performed in the second study year and the second three months are performed during the third year.
“The balance between theoretical and practical training shall, in respect of each subject, give sufficient importance to theory to maintain the university character of the training”	This point was object of intensive discussion during the degree reform according to Bologna. From the university point of view, we need to place emphasis on the theoretical knowledge in order to prepare the students for further studies (Ph.D.).
